# CNT Sheet Air Electrode for the Development of Ultra-High Cell Capacity in Lithium-Air Batteries

**DOI:** 10.1038/srep45596

**Published:** 2017-04-05

**Authors:** Akihiro Nomura, Kimihiko Ito, Yoshimi Kubo

**Affiliations:** 1 GREEN, National Institute for Materials Science, 1-1 Namiki, Tsukuba 305-0044, Japan

## Abstract

Lithium-air batteries (LABs) are expected to provide a cell with a much higher capacity than ever attained before, but their prototype cells present a limited areal cell capacity of no more than 10 mAh cm^−2^, mainly due to the limitation of their air electrodes. Here, we demonstrate the use of flexible carbon nanotube (CNT) sheets as a promising air electrode for developing ultra-high capacity in LAB cells, achieving areal cell capacities of up to 30 mAh cm^−2^, which is approximately 15 times higher than the capacity of cells with lithium-ion battery (LiB) technology (~2 mAh cm^−2^). During discharge, the CNT sheet electrode experienced enormous swelling to a thickness of a few millimeters because of the discharge product deposition of lithium peroxide (Li_2_O_2_), but the sheet was fully recovered after being fully charged. This behavior results from the CNT sheet characteristics of the flexible and fibrous conductive network and suggests that the CNT sheet is an effective air electrode material for developing a commercially available LAB cell with an ultra-high cell capacity.

Due to the increasing demand for vast energy storage media, battery systems far exceeding the current lithium-ion battery (LiB) technologies, such as lithium-sulfur, multivalent ions, or metal-air batteries, have been under development. Lithium-air batteries (LABs), which deliver electric energy from the aerial oxidation of lithium metal, have large theoretical energy densities of up to 3,500 Wh kg^−1^ (based on the weight of lithium peroxide (Li_2_O_2_) as a discharge product), which is more than 10 times that of current LiBs and is the largest among those developing batteries^1^,^2^,^3^,^4^. By leveraging this high energy density nature, LABs are expected to be used to develop a cell with a larger capacity than ever before. Since the pioneering work by Abraham *et al*. on a rechargeable LAB cell in 1996^5^, researchers have focused on assembling practically available LAB cells while concurrently working to fundamentally understand the battery reaction. The cell capacities of actual LAB cells are dominated by the oxygen-breathing cathode (air electrode), in which the discharge product Li_2_O_2_ is deposited/decomposed along with the discharge/charge through the fundamental electrochemical reaction of 2Li^+^ + O_2_ + 2e^−^ ↔ Li_2_O_2_. Several nanocarbons, such as carbon black (such as Super-P^®^ or Ketjenblack^®^)^6^,^7^,^8^, graphene^9^,^10^,^11^,^12^, carbon aerogels^13^, carbon fibers^14^,^15^,^16^, or carbon nanotubes (CNTs)^17^,^18^,^19^,^20^,^21^,^22^, have been proposed as air electrode materials. Some of these nanocarbons exhibit very high specific capacities of ~10,000 mAh g_carbon_^−1^ owing to their huge surface areas of ~1,000 m^2^ g^−1^. However, their lean carbon loadings mostly below 1 mg cm^−2^ limit their resulting cell capacities per electrode area to no more than 10 mAh cm^−2^. Although this areal capacity is 5 times larger than the capacity of the current LiB cells of ~2 mAh cm^−2^, an even larger areal capacity is needed to assemble practical LAB cells with the expected energy density. In fact, enhancing the cell capacity per electrode area (mAh cm^−2^) is essential to developing a practical cell with an extremely large energy density.

Among the proposed nanocarbons, CNTs have a unique character stemming from their nanofibrous structure. Powdery carbon materials require rigid solidification to be handled as air electrodes, but such firm solid electrodes are often not tolerant of the mechanical stress triggered by the discharge solid deposition, especially in deeply discharged conditions. In contrast, CNTs provide paper-like flexible sheets as a result of their tubular constitution that are ready for use as air electrodes in LABs^17^,^18^,^19^,^20^,^21^,^22^. The fibrous network of CNTs is also beneficial for retaining the conductive network in the electrode during the deposition/decomposition of the discharge solid. Here, we report that CNT sheets can be used to attain an ultra-high cell capacity in LAB cells to achieve a much higher areal cell capacity of up to 30 mAh cm^−2^, which is 15 times higher than the capacity of current LiB cells.

## Results and Discussion

Single-walled CNTs (SWCNTs) with metallic conductivity and a relatively small tube diameter of approximately 2 nm (Figure S1) were chosen as the starting material in the fabrication of a CNT sheet air electrode to obtain a surface area that was as large as possible. Two types of CNT sheets were prepared through vacuum filtration of CNTs that had been ultrasonically dispersed in isopropanol (IPA) or *N*-methyl-2-pyrrolidone (NMP). CNT-IPA and CNT-NMP denote the corresponding CNT sheets prepared from the CNT slurries in IPA and NMP, respectively. CNT-IPA and CNT-NMP were composed of a non-woven textile of CNT bundles with ~50 nm and ~150 nm average widths, respectively, based on scanning electron microscopy (SEM) observations (Figure S2) and had 65% and 60% porosity based on the sheet weight-thickness relationships (Figure S3) for CNT-IPA and CNT-NMP, respectively. The LAB cells in the CR2032 configuration (2.0 cm^2^ electrode area) using CNT-IPA or CNT-NMP as air electrodes had a constant discharge voltage of 2.65 V and a gradually increasing charge voltage of up to 4.5 V (Figure S4). The CNT-IPA cell emitted an output current of up to 0.4 mA cm^−2^ (Figure S5), and the cell repeated the discharge/charge cycle up to 73 times with a limited cycle capacity of 0.5 mAh cm^−2^ (Figure S6); all of these cell performance parameters are comparable to those of LAB cells using solidified Ketjenblack^®^^8^ or graphene air electrodes^12^.

**Figure 1:**
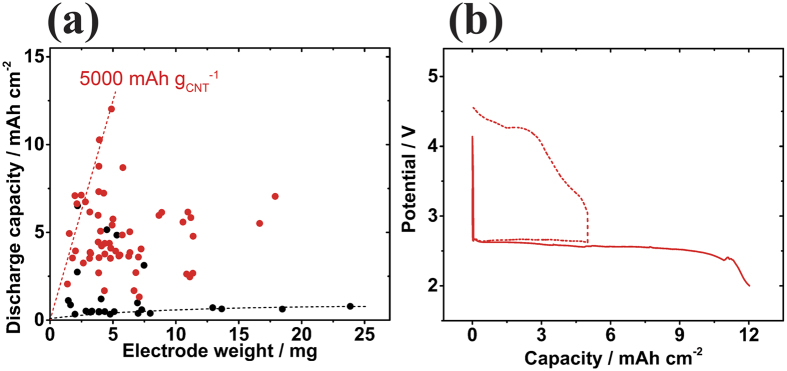
(**a**) Discharge capacities of LAB coin cells with CNT-IPA (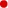) and CNT-NMP (•) air electrodes. The capacity was plotted against the weight of the CNT sheet electrode in a ϕ16 mm disk shape. The red dotted straight line shows the capacity per CNT electrode weight of 5,000 mAh g_CNT_^−1^. A maximum discharge capacity of 12 mAh cm^−2^ (24 mAh per cell) was obtained using a cell with a 5 mg CNT-IPA electrode. (**b**) Discharge profile of the CNT-IPA cell that has a discharge capacity of 12 mAh cm^−2^ (solid line). A typical discharge-charge cycle profile of the cell at a fixed cycle capacity of 5 mAh cm^−2^ is shown by the dotted line.

Figure 1(a) shows the discharge capacities of cells possessing different CNT sheet thicknesses before reaching the cutoff voltage of 2.0 V. The capacities were plotted against the electrode weight, which is directly proportional to the sheet thickness. Although a negligible difference in battery performance was observed between the CNT-IPA and CNT-NMP cells in the limited cycle capacity operation (Figure S4), the CNT-NMP cells had smaller discharge capacities not exceeding 1 mAh cm^−2^ regardless of their thickness, excluding a few outliers, owing to the difference in the CNT bundle width and hence the inhomogeneous occurrence of the LAB reaction inside the CNT-NMP, which will be discussed later. In contrast, most of the CNT-IPA cells had much higher discharge capacities. Though their capacities varied widely with the differences in each cell, the maximum discharge capacity at each electrode weight linearly increased with the sheet weight up to 5 mg (56 μm thick), illustrating the efficiency of the sheet electrode at a discharge capacity of 5,000 mAh g_CNT_^−1^ (linear dotted line). As a result, the CNT-IPA cell achieved a maximum discharge capacity of 12 mAh cm^−2^ (24 mAh per cell), which was 6 times higher than the capacity of the conventional LiB cells of approximately 2 mAh cm^−2^. Figure 1(b) shows the discharge profile. Even though the efficiency of the discharge capacity (5,000 mAh g_CNT_^−1^) is not outstanding compared to the efficiencies of all reported air electrode materials, the maximum cell capacity of 12 mAh cm^−2^ is quite competitive. The rechargeability of the cell was also confirmed, as shown by the dotted line in Fig. 1(b). However, the maximum capacity was not further improved by simply increasing the sheet thickness, which implies that the sheet electrode was becoming suffocated as the thickness was increased.

**Figure 2:**
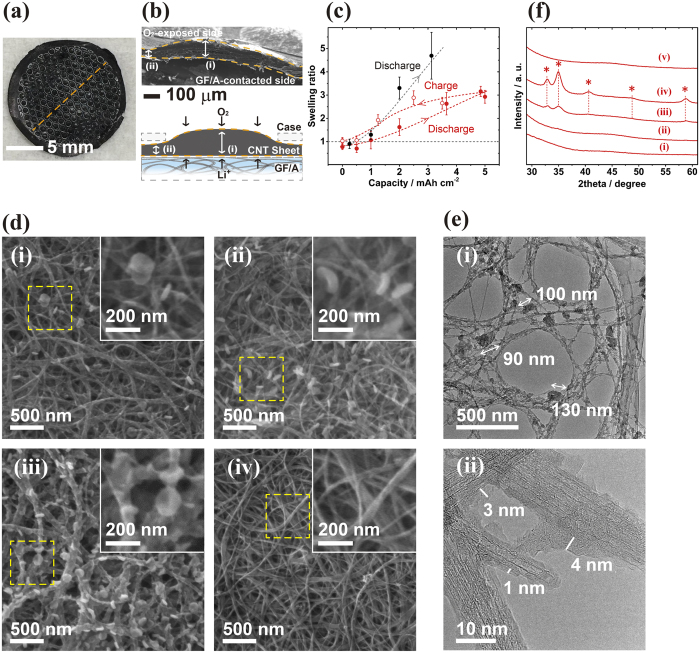
(**a**) Photographic image of a typical CNT sheet after the discharge experiment. The sheet was cut with precision scissors along the orange dotted line for cross-sectional SEM observation. (**b**) Typical cross-sectional SEM image of the CNT sheet after the discharge experiment (up) and its schematic illustration (low). The orange dotted line highlights the edges of the cross section. (**c**) Swelling ratios of CNT-IPA (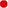) and CNT-NMP (•) after discharge. The ratios of CNT-IPA after charging from a 5 mAh cm^−2^ discharged state were plotted as open red circles (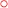). (**d**) SEM images of CNT-IPA after discharges of 1 mAh cm^−2^ (i), 2 mAh cm^−2^ (ii), and 5 mAh cm^−2^ (iii) and after being fully charged following a 5 mAh cm^−2^ discharge (iv). The dotted yellow squares show the magnified areas superimposed in each image. (**e**) Low- (i) and high- (ii) magnification TEM images of CNT-IPA after a 9 mAh cm^−2^ discharge. (**f**) XRD spectra of pristine CNT-IPA (i) and after discharges of 1 mAh cm^−2^ (ii), 2 mAh cm^−2^ (iii), and 5 mAh cm^−2^ (iv) and after being fully charged following a 5 mAh cm^−2^ discharge (v). The *symbols denote Li_2_O_2_ crystal reflections of 100, 101, 102, 004, and 110 at 2theta ≈32.9, 35.0, 40.6, 48.7, and 58.6, respectively.

After the discharge experiment, the CNT sheet was observed to have expanded considerably by extruding into the 0.8 mm-diameter holes perforated on the stainless-steel case of the cathode for oxygen intake (Fig. 2(a)). The cross-sectional SEM image of the sheet (Fig. 2(b)) revealed that the part of the CNT sheet that faced the open perforated holes (b,i) experienced the increase in sheet thickness, but the part harnessed by the cathode case (b,ii) did not expand from its initial thickness. Figure 2(c) shows the swelling ratio of the initial sheet thickness (~50 μm) at the protruding parts (b,i) of CNT-IPA and CNT-NMP during the discharge/charge cycle. In the discharge process, CNT-IPA began to swell at 1 mAh cm^−2^ discharge and kept swelling to an approximately 3-fold thickness (~150 μm) at 5 mAh cm^−2^ discharge. In the charge process, the sheet thickness of CNT-IPA monotonously shrank with increasing charge, returning to its initial thickness in the fully charged state. The CNT-NMP sheets abruptly distended by 4.8 times at 3 mAh cm^−2^ discharge with their extending error bars. The large error values for CNT-NMP resulted from the considerable roughness and layered voids created inside the sheet. The cross-sectional SEM observation and the corresponding energy-dispersive X-ray spectroscopy (EDS) analysis of CNT-IPA and CNT-NMP revealed that the battery reaction occurred inhomogeneously inside CNT-NMP (Figure S7), which explains why the most of the CNT-NMP cells stopped discharging at low discharge capacities; the inhomogeneous reaction and hence the localized deposition of the discharge product suffocated the rest of the sheet, rendering it unusable for the discharge reaction and resulting in lower discharge capacities in the CNT-NMP cells.

The sheet was subjected to microscopic analysis after the discharge/charge experiment to investigate what triggered the swelling/shrinkage of the sheet. Figure 2(d) shows magnified SEM images of CNT-IPA after the discharge/charge experiment. The images obtained after discharge illustrate the deposition process of the discharge product; first, disk particles 50–150 nm in diameter were deposited on CNT bundles, and the number of particles then increased, with the particles evolving into a toroidal shape. Additionally, the bundles slightly thickened as the discharge progressed. The characteristic toroid shape suggests the formation of Li_2_O_2_ as a discharge product, which has been reported in a wide range of LAB cells with non-carbonate electrolytes and carbonaceous air electrodes^7^,^11^,^12^,^15^,^23^,^24^,^25^,^26^,^27^, and the detailed mechanism of the morphological evolution has been discussed by Yang and Thompson^21^. The full charge from a 5 mAh cm^−2^ discharged state completely decomposed the discharge product, restoring it to the initial bundle network structure. Transmission electron microscopy (TEM) observations of the CNT bundles after discharge (Fig. 2(e)) revealed the presence of a filmy product with a thickness of 5 nm or less covering the CNT bundles, in addition to the particle deposits of 50–150 nm observed in the SEM images. The deposition/decomposition of the discharge product in these toroidal or filmy morphologies caused the CNT sheet to reversibly swell/shrink, maintaining the conductive fibrous network of CNT bundles. X-ray diffraction (XRD) analysis of the CNT sheets after the discharge/charge experiment (Fig. 2(f)) showed peaks that were identically attributed to Li_2_O_2_ crystal diffraction after 2 mAh cm^−2^ discharge. As no other peaks aside from Li_2_O_2_ were found, Li_2_O_2_ was the exclusive discharge product of the discharge/charge reaction of the LAB. The peaks increased in intensity with further discharge to 5 mAh cm^−2^ but completely disappeared after reaching full charge, which is consistent with the toroid evolution observed by SEM.

**Figure 3:**
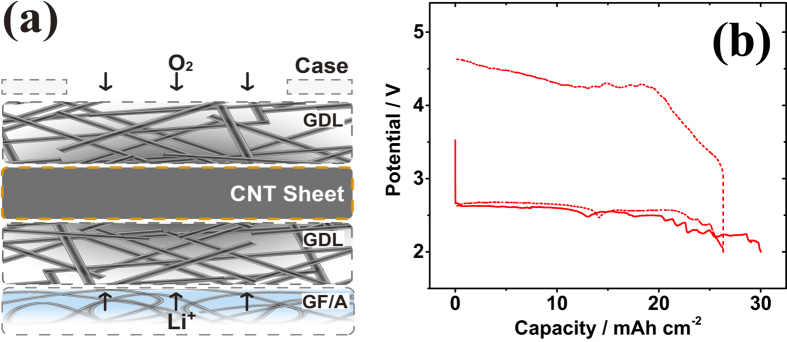
(**a**) Schematic illustration of the CNT-GDL composite air electrode. (**b**) Discharge profile of the cell with a CNT-GDL composite air electrode that discharged to 30 mAh cm^−2^ (60 mAh per cell, red solid line). The dotted line shows the typical discharge-charge cycle profile of the cell at a capacity of 26 mAh cm^−2^.

These microscopic analyses revealed that the large discharge capacity of the cell with the CNT sheet air electrode was a result of the flexible sheet characteristic with discharge-dependent swellability. The fixed position of the CNT sheets inside the cell cases raises concern that the attained discharge capacity might have been suppressed by the limited sheet swelling within the range of the windows of the cathode case holes. In an attempt to extend the swelling range of the CNT sheet during discharge, a gas diffusion layer (GDL), an array of solid carbon fibers ~10 μm in diameter (TGP-H-060, 190 μm thickness, Toray, Japan), was placed on both sides of the CNT sheet air electrode, which dramatically increased the discharge capacity relative to the cells without GDL insertion. Figure 3(a) shows a schematic illustration of the GDL-inserted air electrode. The red solid line in Fig. 3(b) shows a discharge profile of a cell with the air electrode of CNT-IPA sandwiched between two GDLs, recorded before reaching the cutoff voltage of 2.0 V. The cell achieved a 30 mAh cm^−2^ discharge capacity (60 mAh attained per cell), which is 15 times higher than that of conventional LiB cells. The authors believe that this ultra-high discharge capacity was a result of the inserted GDLs, which provided the CNT sheet with additional space for swelling during discharge. Importantly, the cell capacity purely resulted from the CNT sheet, as the GDL itself contributed little, less than 0.05 mAh cm^−2^ by a single GDL, to the discharge capacity. The cell with the CNT-GDL composite air electrode was also fully rechargeable, as shown by the dotted line in Fig. 3(b). Although the cyclic performance of this cell is being studied by our group, further studies on the anode and electrolyte are required to collect subsequent discharge/charge cycle profiles. The behaviors of the Li metal and the TEGDEM electrolyte during such long-term operation are currently unknown. The bumpy discharge voltage observed in the discharge profiles reflects the progress of the swelling of the CNT sheet inside the GDL. To the best of our knowledge, this cell capacity is the highest among the prototype LAB cells reported to date.

**Figure 4:**
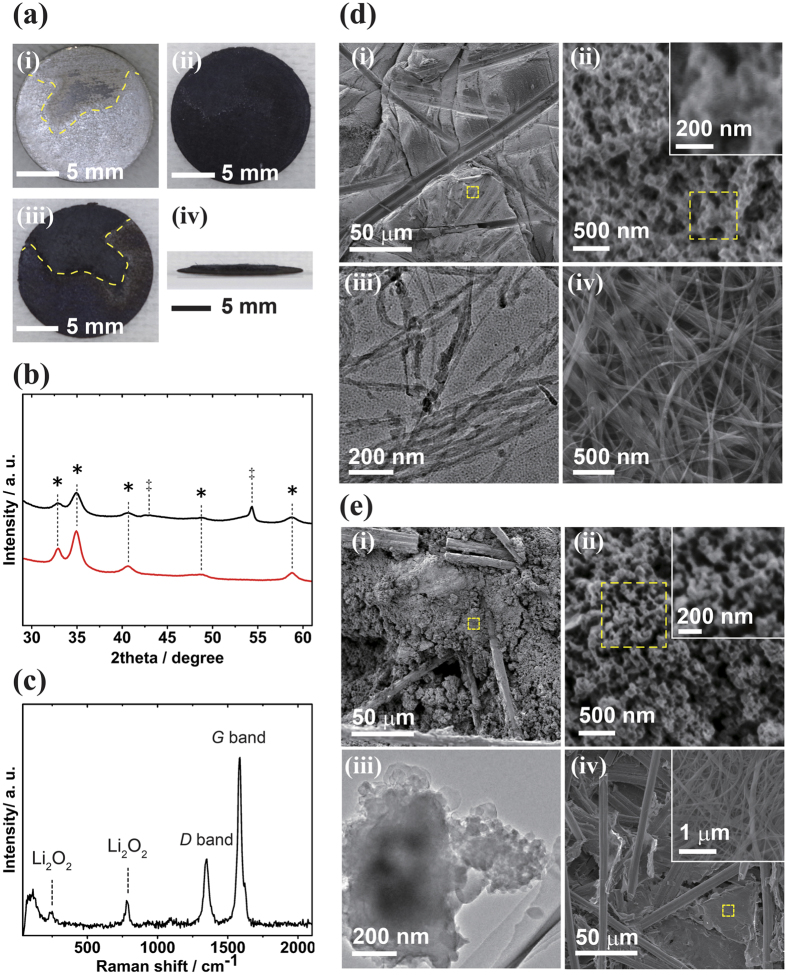
(**a**) Photographic images of each electrode of the CNT-GDL cell after a 20 mAh cm^−2^ discharge. Lithium metal anode (i), CNT sheet (ii), and GDL (iii) with its edge-on image (iv). The yellow dotted lines in (i) and (iii) highlight the depleted lithium metal and the sections of protruded deposit on the GDL surface, respectively. (**b**) XRD spectra of the CNT sheet (red line) and the GDL (black line) after a 20 mAh cm^−2^ discharge. The *symbols denote Li_2_O_2_ crystal reflections, as shown in Fig. 2(f). The ‡symbols denote graphite reflections of 101 and 004 at 2theta ≈42.9 and 54.4, respectively. (**c**) Raman spectrum of the GDL after a 20 mAh cm^−2^ discharge. The two intense peaks are attributed to the *D* (1350 cm^−1^) and *G* (1580 cm^−1^) bands of graphitic carbon fiber. The other two peaks at 260 and 790 cm^−1^ belong to Li_2_O_2_ vibrations. (**d**) SEM images of the CNT sheet after 20 mAh cm^−2^ discharge ((i) low and (ii) high magnification). The yellow dotted squares show the magnified areas. TEM image of the CNT bundles (iii). SEM image of the CNT sheet after being fully charged following a 15 mAh cm^−2^ discharge (iv). (**e**) SEM images of the GDL after a 20 mAh cm^−2^ discharge ((i) low and (ii) high magnifications). The yellow dotted squares show the magnified areas. TEM image of the discharge product deposited inside the GDL (iii). SEM image of the GDL after being fully charged following a 15 mAh cm^−2^ discharge (iv).

The cell with the CNT-GDL composite air electrode was disassembled after discharge, which revealed the enormous amount of CNT swelling over the GDL as the result of Li_2_O_2_ deposition. Figure 4(a) shows photos of each electrode from the cell discharged at 20 mAh cm^−2^. The lithium metal anode (200 μm thickness) was partly depleted, revealing the stainless-steel anode case at the bottom (a, i), and this depletion could have stopped the discharge. Further increases in the discharge capacity to 40 mAh cm^−2^ (80 mAh per cell) can be obtained by using thicker lithium metal (1 mm), but these data will be reviewed in a subsequent report. The CNT sheet was turned into a crisp solid after 20 mAh cm^−2^ discharge, implying that the CNT bundle network was completely filled with the discharge product (a, ii). The CNT sheet thickness was approximately 150 μm (swelling ratio ~3) at 15 mAh cm^−2^ discharge. The inserted GDL had a black mound on the surface (a, iii) in the area where the lithium anode had been depleted. The mound was 1–2 mm thick when viewed edge-on (a, iv), corresponding to ~10-fold swelling from the pristine GDL (initially 190 μm thickness). Since the GDL itself contributes little to the discharge reaction, the black mound generated on the GDL surface is deemed to be the discharge product grown from the CNT bundles that had penetrated inside the GDL. The intruding CNT bundles into the GDL are discussed later. Additionally, the deposit mound was observed on the GDL that faced the separator, and little deposition appeared on the air side.

XRD (Fig. 4(b)) and Raman spectroscopy (Fig. 4(c)) analyses of the CNT-GDL composite air electrode revealed that Li_2_O_2_ was the exclusive discharge product on both the CNT and GDL parts, even in an ultra-high discharge state, because their spectra contained peaks that can be attributed only to Li_2_O_2_, except for the carbon graphite substrate. The SEM observation of the CNT part after discharge (Fig. 4(d)) displayed several grooves corresponding to traces of carbon fibers from the GDL (d, i), and the magnified image of the sheet surface showed heavily beaded particles of ~100 nm around the CNT bundles (d, ii), implying that no additional space existed to accommodate Li_2_O_2_ particles on the bundle surface. The TEM image of the sheet (d, iii) also showed the beaded deposits on the CNT bundles observed the SEM image, and even from the densely packed state, the sheet was recovered to the non-woven bundle textile state at full charge (d, iv). On the other hand, the SEM observation of the black mound on the GDL (Fig. 4(e)) indicates a densely filled deposit between the gaps in the carbon fibers of the GDL (e, i). The magnified image of the deposit revealed granular particles of ~100 nm captured by thin fibers (e, ii), and the TEM observation revealed fine aggregates of the particles (e, iii). The presence of CNT bundles that had seeped into the GDL was clearly confirmed by the image of the GDL after being fully charged, depicting CNT bundles remaining inside the carbon fibers (e, iv).

The evidence supporting Li_2_O_2_ as the exclusive discharge product indicates that the ideal LAB reaction nominally written as 2Li^+^ + O_2_ + 2e^−^ → Li_2_O_2_ is occurring even during the discharge to ultra-high capacity. The 30 mAh cm^−2^ discharge capacity achieved by the cell with the CNT-GDL composite air electrode consumed 150 μm of the thickness of the Li metal anode to form 120 μm of bulk thickness of Li_2_O_2_ on the cathode. This thickness of Li_2_O_2_ could barely be held by the rigid carbon cathode but was successfully stored in the flexible CNT sheet by the swelling of the sheet to a thickness of a few millimeters. The CNT sheet (CNT-IPA, ~50 nm bundle width) had a calculated surface area of 60 m^2^g^−1^. Although this surface area is much less than that of the rigid carbon cathode, the swellable nature of the sheet provides distinctive advantage to LAB cells for extending their areal cell capacity. The 120 μm-thick Li_2_O_2_ deposit on CNT-IPA numerically leads to ~80 nm-thick Li_2_O_2_ on the CNT bundle surface, which is close to the particle size observed by SEM and TEM in Figs 2 and 4. However, the detailed reaction mechanism by which the Li_2_O_2_ discharge product grows is not clear at this time. “Plating” and “precipitation” processes have been discussed for Li_2_O_2_ growth in LABs^28^. In the “plating” process, which is considered a two-electron reaction, the LAB reaction occurs only on the cathode surface to form Li_2_O_2_, but the insulative nature of Li_2_O_2_ limits its growth to only a few nm in thickness. This process indicates that the filmy deposit on the CNT bundles observed in Fig. 2(e, ii) was Li_2_O_2_ but would hardly be able to produce particles with thicknesses of ~100 nm. On the other hand, the “precipitation” process involves a one-electron reaction in an electrolyte to form solvated LiO_2_ (Li^+^ + O_2_ + e^−^ → LiO_2_), which is subsequently precipitated (disproportionated) on the cathode surface as Li_2_O_2_ while producing O_2_ (2LiO_2_ → Li_2_O_2_ + O_2_). Contaminated protons promote the solvation of LiO_2_, and micron-sized toroidal Li_2_O_2_ shapes have been observed on the cathode in an electrolyte containing a few thousand ppm H_2_O^29^,^30^. The presence of small particles with ~100 nm thickness in Figs 2 and 4 suggests that Li_2_O_2_ was generated through the precipitation process in this study. However, the experiments were performed in a dry state (H_2_O < 30 ppm), and it is uncertain whether this process can provide the mechanical stress needed to swell the CNT sheet to a thickness of a few millimeters. Since the mechanism of Li_2_O_2_ growth is directly related to the design of the air electrode for deriving cell performance, further study is in progress to determine the Li_2_O_2_ deposition process.

In summary, LAB cells with CNT sheet air electrodes were prepared to extend the actual cell capacity of the LAB cell, exploiting the sheet characteristics of the flexible and fibrous network. The CNT sheet air electrode swelled along with the discharge and resulted in LAB cells that can discharge up to 30 mAh cm^−2^. This discharge capacity is 15 times higher than that of current LiB cells and is the highest among the prototype LAB cells reported to date, to the best of our knowledge. As a result, the sheet electrode experienced enormous expansion owing to deposition of the discharge product on the CNT bundles and grew into an expanded cathode with a thickness of a few millimeters. The discharge product deposit was solely identified as Li_2_O_2_ based on XRD and Raman analyses, which implies that the ideal LAB reaction was occurring inside the cathode during the discharge. Therefore, the CNT sheet is a promising air electrode material for achieving an ultra-high cell capacity in LAB cells. In addition, the invention of long-lasting air electrode provide paths for studying appropriate designs of Li metal anodes and electrolytes for LAB. These push new frontier for constructing practically available LAB cells in the future.

## Methods

### Preparation of the CNT Sheet Air Electrode

Seventy-five milligrams of SWCNTs with an average tube diameter of 2 nm (EC2.0, Meijo Nano Carbon) was dispersed in 150 g of IPA or NMP with an ultrasonic homogenizer (450D, Branson) equipped with a 1/8” micro-tip for 3 h in an ice bath. The prepared slurry of SWCNTs was filtered under vacuum through a PTFE membrane (Omnipore^TM^, 1 μm pore size, EMD Millipore) to obtain a free-standing and binder-free CNT sheet. After drying in a vacuum oven at 60 °C overnight, the CNT sheet was deliberately peeled off from the membrane and cut into a 16 mm-diameter cathode (air electrode, 2 cm^2^ effective area). Air electrodes of various thickness (10–250 μm thick) were prepared by adjusting the filtered amount of the slurry.

### Battery Assembly and Testing

Batteries were assembled in a CR2032 coin cell with perforated stainless-steel cases (Hohsen Corp.) in a dry room with a supply-air dew point of <−90 °C (<0.1 ppm of H_2_O) and an environmental dew point of approximately −60 to −50 °C. The cell comprised layers of a Li metal foil anode (16 mm in diameter, 200 μm thick, Honjo Metal), glass fiber separator (GF/A, Whatman), and CNT sheet air electrode facing the perforated side of the cell. Two films of a PP/PE/PP trilayer porous membrane (20 μm thick) were placed on both sides of the GF/A separator to prevent the glass fibers from sticking to the anode and air electrode. An ether-based electrolyte, composed of 1 M lithium bis(trifluoromethanesulfonyl)imide (LiTFSI, Kishida Chemical Co., Ltd.) dissolved in anhydrous tetraethylene glycol dimethyl ether (TEGDME, <10 ppm H_2_O, Japan Advanced Chemicals) was prepared in an Ar-filled glovebox (Glovebox Japan Inc.) and immersed in the GF/A glass fiber separator and the CNT sheet air electrode. The prepared electrolyte had a water content of less than 30 ppm, determined by Karl Fisher titration before the immersion. Discharge and charge profiles were collected under oxygen gas flow at a constant current density of 0.1 mA per cell (0.05 mA cm^−2^) using a battery discharge-charge system (HJ1001SD8, Hokuto Denko). The lower limit of the discharge voltage was set at 2.0 V, where the LAB normally reaches its fully discharged state.

### Characterization

After the discharge/charge experiment, the batteries were disassembled in the dry room to collect the CNT sheet air electrode for analysis. The collected CNT sheets were copiously rinsed with super dehydrated tetrahydrofuran (<10 ppm H_2_O, Wako Pure Chemicals) and then dried under vacuum. The completely dried CNT sheets were transported using air-isolated sample holders to prevent possible reactions with the humidity in ambient air. The morphologies and structures of the CNT sheet air electrode were analyzed using a JSM-7800F field-emission scanning electron microscope (FE-SEM, JEOL) with a 5 keV accelerating voltage. EDS was performed using an X-Max^N^ analyzer (Oxford Instruments) attached to the SEM. TEM images of the CNTs and discharge product were obtained from a JEOL JEM-ARM 200 F at 200 or 80 keV accelerating voltage. XRD measurements were carried out on a D8 ADVANCE (Bruker) powder X-ray diffractometer with a CuKα source (λ_CuKα_ = 1.542 Å).

## Additional Information

**How to cite this article**: Nomura, A. *et al*. CNT Sheet Air Electrode for the Development of Ultra-High Cell Capacity in Lithium-Air Batteries. *Sci. Rep.* **7**, 45596; doi: 10.1038/srep45596 (2017).

**Publisher's note:** Springer Nature remains neutral with regard to jurisdictional claims in published maps and institutional affiliations.

## Supplementary Material

Supplementary Information
