# Supplementary material for: CNT Sheet Air Electrode for the Development of Ultra-High Cell Capacity in Lithium-Air Batteries
Source: Sci Rep. 2017 Apr 5;7:45596. doi: 10.1038/srep45596 (PMC5381228; doi:10.1038/srep45596)
Supplement: Supplementary Information [file srep45596-s1.pdf]

Supplementary Information

CNT Sheet Air Electrode for the Development of Ultra-High Cell Capacity in Lithium-Air Batteries

Akihiro Nomura, Kimihiko Ito, and Yoshimi Kubo*

GREEN, National Institute for Materials Science, 1-1 Namiki, Tsukuba 305-0044, Japan

"*Correspondence and requests for materials should be addressed to Yoshimi Kubo (email: KUBO.Yoshimi@nims.go.jp)"

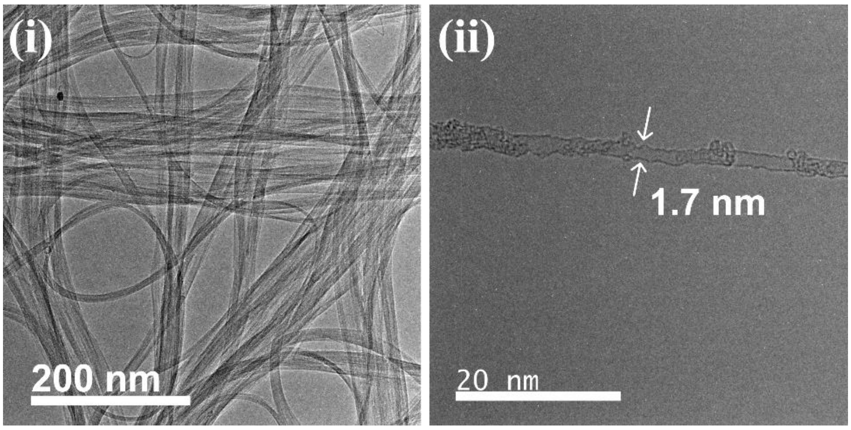

Supplementary Figure S1. Low- (i) and high- (ii) magnification TEM images of the SWCNTs used in this study.

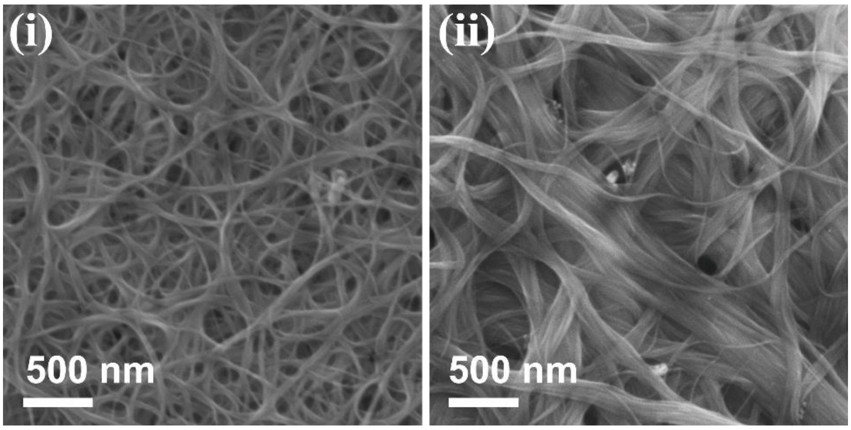

Supplementary Figure S2. SEM images of CNT-IPA (i) and CNT-NMP (ii). Although the same SWCNTs were used to obtain CNT-IPA and CNT-NMP, the higher boiling temperature and hence longer drying time required for NMP compared to IPA might have resulted in the thicker bundles in CNT-NMP (~150 nm width) compared to CNT-IPA (~50 nm width) observed during the preparation, with greater chance for re-aggregation.

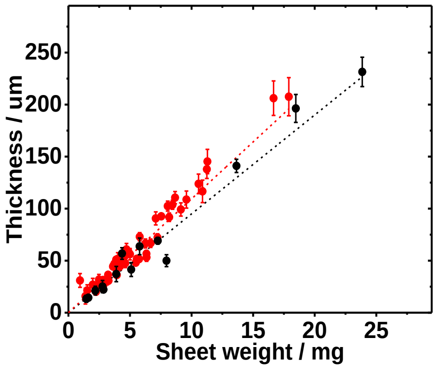

Supplementary Figure S3. Thickness of CNT-IPA (●) and CNT-NMP (●) plotted against the weight for a disk shape 16 mm in diameter. The dotted lines represent the linear regression results for each sheet, and the slopes of the lines provide sheet densities of 0.45 g cm^{-3} and 0.52 g cm^{-3} for CNT-IPA and CNT-NMP, respectively. Using the weight densities of these sheets and the actual density of the SWCNTs (1.3 g cm^{-3}, catalogue value), the porosities of the CNT sheets were estimated as 65% and 60% for CNT-IPA and CNT-NMP, respectively.

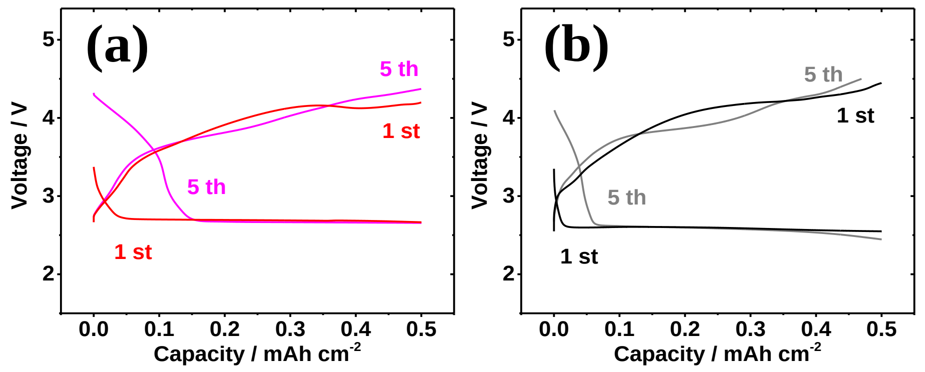

Supplementary Figure S4. Typical discharge-charge cycle profiles of the LAB cells with 10.9 mg of the CNT-IPA air electrode (a) and 7.3 mg of the CNT-NMP air electrode (b). Both profiles were recorded at a fixed cycle capacity of 0.5 mAh cm^{-2} and a current of 0.05 mA cm^{-2}. The profiles only depict the discharge/charge cycle of the 1st and 5th runs.

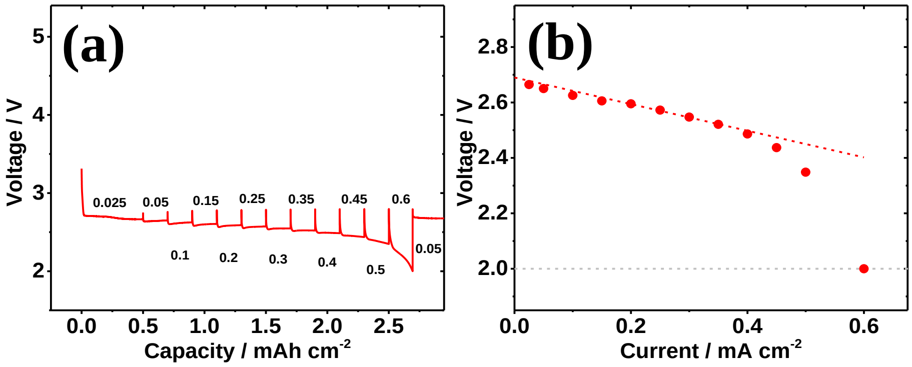

Supplementary Figure S5. (a) Typical discharge profile of LAB coin cells with the CNT-IPA air electrode at varied discharge currents of 0.025-0.6 mA cm^{-2}. The inset numbers are the discharge current values in mA cm^{-2}. A fixed capacity of 0.2 mAh cm^{-2} was discharged at each discharge current before reaching a cutoff voltage of 2.0 V. (b) Terminal voltages at each discharge current interval in (a) plotted against their discharge currents. The voltage linearly decreased as the discharge current increased to 0.4 mA cm^{-2}, suggesting that the discharge voltage was dominated by iR drops, following V_{dis}-iR, where V_{dis}, i, and R represent the equilibrium potential, discharge current, and internal resistance of the CNT-IPA cells, respectively. The slopes of the dotted straight lines gave R values of 240 Ω, and the extrapolation of the line to a discharge current of 0 mA cm^{-2} gave a V_{dis} value of 2.69 V. The difference between the V_{dis} and standard thermodynamic potential of 2.96 V of the LAB resulted from deviations in the running temperature, electrolyte concentration, and electrode surfaces.

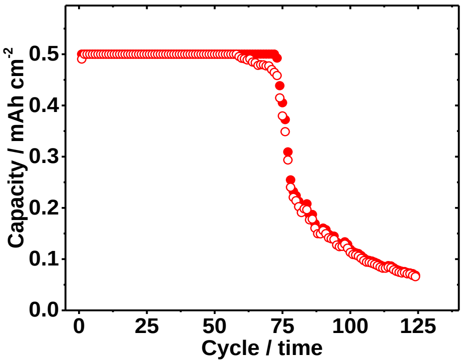

Supplementary Figure S6. Discharge (●) and charge (○) capacities vs. cycle number of the CNT-IPA cell cycled at a fixed capacity of 0.5 mAh cm^{-2} and a current of 0.05 mA cm^{-2}.

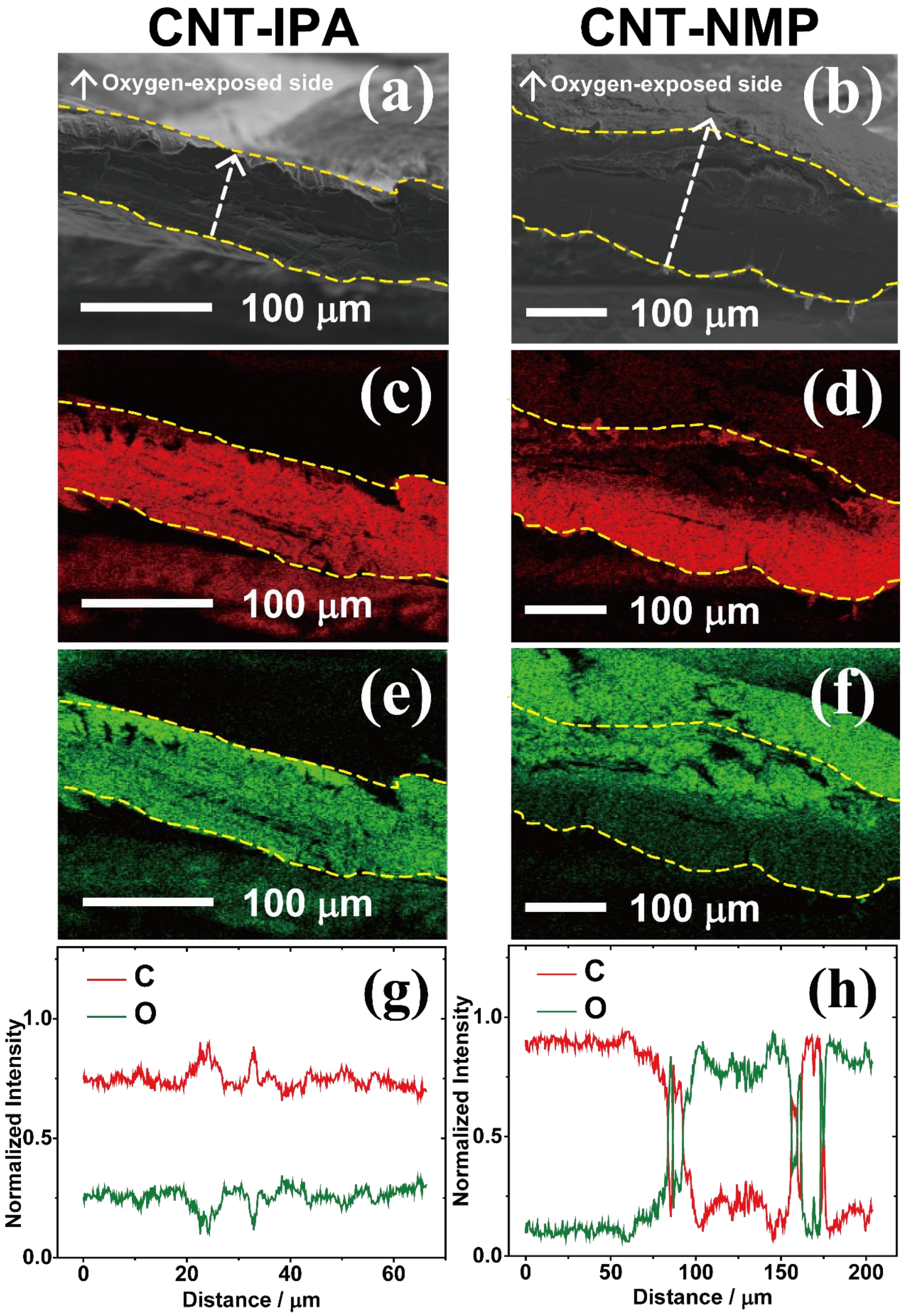
CNT-IPA
CNT-NMP
Oxygen-exposed side
(a)
100 μm
Oxygen-exposed side
(b)
100 μm
(c)
100 μm
(d)
100 μm
(e)
100 μm
(f)
100 μm
C
O
(g)
Normalized Intensity
0.0
0.5
1.0
0
20
40
60
Distance / μm
C
O
(h)
Normalized Intensity
0.0
0.5
1.0
0
50
100
150
200
Distance / μm

Supplementary Figure S7. (a, b) Cross-sectional SEM image of the CNT-IPA (a) and CNT-NMP (b) air electrodes after a 2 mAh cm^{-2} discharge. The yellow dotted lines in the images show the edges of their cross-sections. The upper side of the sheets faced the oxygen flow during the discharge experiment. (c-f) Corresponding EDS mappings of carbon (C, (c, d)) and oxygen (O (e, f)) in the SEM images of (a) and (b). The C and O elemental peaks originate from the CNTs and discharge product deposit, respectively. The distribution of C and O represents the extent to which the LAB reaction occurred inside the CNT sheets; the area with a higher C content corresponds to the place where less battery reaction occurred, whereas a higher O content indicates an area where the battery reaction severely occurred. (g, h) EDS line scan profiles of C and O through section denoted by the white dotted line in the SEM images of (a) and (b). Their intensities were normalized by the total intensity of C and O. The uniform distribution of C and O across the CNT-IPA (g) indicates that the battery reaction occurred evenly throughout the sheet during discharge. Meanwhile, the uneven distribution of O on oxygen-exposed side (h) indicates that the regional battery reaction mainly occurred on the side of the sheet in contact with O. Even in CNT-IPA, the inhomogeneous deposition behavior became gradually obvious with further discharge over 5 mAh cm^{-2}, which partly explains the scattered discharged capacities in Figure 1 (a), where the uneven deposit blocked the continuous diffusion of the battery reactants to stop further discharge at unpredictable discharge capacities.
